# Moving beyond size: vorticity and energy loss are correlated with right ventricular dysfunction and exercise intolerance in repaired Tetralogy of Fallot

**DOI:** 10.1186/s12968-021-00789-2

**Published:** 2021-08-19

**Authors:** Yue-Hin Loke, Francesco Capuano, Vincent Cleveland, Jason G. Mandell, Elias Balaras, Laura J. Olivieri

**Affiliations:** 1 Present Address: Division of Cardiology, Children’s National Medical Center, 111 Michigan Ave NW, W3-200, Washington, DC 20010 USA; 2 Department of Mechanics, Mathematics and Management, Polytechnic University of Bari, Bari, Italy; 3 Sheikh Zayed Institute for Pediatric Surgical Innovation, Children’s National Medical Center, 111 Michigan Ave NW, Washington, DC 20010 USA; 4 Department of Mechanical and Aerospace Engineering, George Washington University, Washington, DC 20052 USA

**Keywords:** Tetralogy of Fallot, Intracardiac flow, Vorticity, 4D flow

## Abstract

**Background:**

The global effect of chronic pulmonary regurgitation (PR) on right ventricular (RV) dilation and dysfunction in repaired Tetralogy of Fallot (rTOF) patients is well studied by cardiovascular magnetic resonance (CMR). However, the links between PR in the RV outflow tract (RVOT), RV dysfunction and exercise intolerance are not clarified by conventional measurements. Not all patients with RV dilation share the same intracardiac flow characteristics, now measurable by time resolved three-dimensional phase contrast imaging (4D flow). In our study, we quantified regional vorticity and energy loss in rTOF patients and correlated these parameters with RV dysfunction and exercise capacity.

**Methods:**

rTOF patients with 4D flow datasets were retrospectively analyzed, including those with transannular/infundibular repair and conduit repair. Normal controls and RV dilation patients with atrial-level shunts (Qp:Qs > 1.2:1) were included for comparison. 4D flow was post-processed using IT Flow (Cardioflow, Japan). Systolic/diastolic vorticity (ω, 1/s) and viscous energy loss (VEL, mW) in the RVOT and RV inflow were measured. To characterize the relative influence of diastolic vorticity in the two regions, an RV Diastolic Vorticity Quotient (ω_RVOT-Diastole_/ω_RV Inflow-Diastole,_ RV-DVQ) was calculated. Additionally, RVOT Vorticity Quotient (ω_RVOT-Diastole_/ω_RVOT-Systole_, RVOT-VQ) and RVOT Energy Quotient (VEL_RVOT-Diastole_/VEL_RVOT-Systole_, RVOT-EQ) was calculated. In rTOF, measurements were correlated against conventional CMR and exercise stress test results.

**Results:**

58 rTOF patients, 28 RV dilation patients and 12 controls were included. RV-DVQ, RVOT-VQ, and RVOT-EQ were highest in rTOF patients with severe PR compared to rTOF patients with non-severe PR, RV dilation and controls (p < 0.001). RV-DVQ positively correlated with RV end-diastolic volume (0.683, p < 0.001), PR fraction (0.774, p < 0.001) and negatively with RV ejection fraction (− 0.521, p = 0.003). Both RVOT-VQ, RVOT-EQ negatively correlated with VO_2-max_ (− 0.587, p = 0.008 and − 0.617, p = 0.005) and % predicted VO_2-max_ (− 0.678, p = 0.016 and − 0.690, p = 0.001).

**Conclusions:**

In rTOF patients, vorticity and energy loss dominate the RVOT compared to tricuspid inflow, correlating with RV dysfunction and exercise intolerance. These 4D flow-based measurements may be sensitive biomarkers to guide surgical management of rTOF patients.

## Background

In repaired Tetralogy of Fallot (rTOF) patients, the long-term consequences of chronic pulmonary regurgitation (PR) on right ventricular (RV) dilation, RV dysfunction, ventricular arrhythmias and mortality have been well-studied [1]. Thus, measurements of RV size and function as guided by cardiovascular magnetic resonance (CMR) have provided critical interventional thresholds for pulmonary valve replacement (PVR) [2, 3]. However, current guidelines acknowledge the limitations of these conventional CMR measurements [2, 3], as they do not necessarily predict deterioration of the RV [4], nor do they incorporate the diversity of RV morphology, RV motion, and RV outflow tract (RVOT) geometry [5–7]. Exclusive interpretation of the RV in rTOF using indexed RV end-diastolic volume (RVEDVI), indexed RV end-systolic volume (RVESVI), RV ejection fraction (RVEF) and pulmonary regurgitant fraction (PR%) does not fully describe RV dysfunction, nor correlate with exercise intolerance [8–11]. Furthermore, the direct link between PR, RV dysfunction and exercise intolerance has not been clarified, as the resultant intracardiac flow abnormalities from PR have not been comprehensively studied in rTOF patients.

Recent advances in CMR now allow for time-resolved three-dimensional phase contrast imaging (4D flow) of intracardiac flow phenomena. With 4D flow, it is observed that intracardiac flow parameters such as vorticity (the local spinning motion of blood) and kinetic energy are altered in rTOF patients [12–15]. Intracardiac flow parameters are thus promising hemodynamic biomarkers to potentially detect RV dysfunction, although these studies have mainly correlated 4D flow biomarkers with PR and RV dilation. These studies have also focused on global quantification of the RV, without considering regional abnormalities created from PR in the RVOT. There have also not been any 4D flow studies that have compared rTOF against other forms of chronic volume loading in the RV.

Beyond size, dilated RVs do not all share the same intracardiac flow characteristics. Both in-vivo and in-vitro studies have highlighted the unique pathophysiology of rTOF patients [16–20] that are fundamentally distinct from other forms of RV dilation. A recent echocardiographic case–control study by Moceri et al. [17] demonstrated different RV strain patterns between rTOF and patients with atrial septal defects (ASD), despite similar RV size. We have previously developed an in-vitro computational model of intracardiac flow in rTOF patients using conventional CMR imaging [18–20]. Through this computational model, we observed a pattern where the jet of PR disrupts the natural tricuspid inflow vortex at the RVOT, a finding also observed in an *in-vitro* pump study by Mikhail et al. [16]. We conjecture that in rTOF patients this flow phenomenon can be quantified by 4D flow as patterns of vorticity and energy loss that are distinct from the normal RV and RV dilation patients.

Thus, in our study, we quantified regional vorticity and energy loss in rTOF patients, and compared these values to normal controls and RV dilation patients (those with dilated RVs from atrial level shunts). We hypothesized that in rTOF patients, there is distinct influence of vorticity and energy loss in the RVOT compared to normal control and RV dilation patients, and that these intracardiac flow parameters are correlated with RV dysfunction and exercise intolerance.

## Methods

This was an Institutional Review Board-approved retrospective study involving patients who underwent a CMR study between December 2018 and August 2020. rTOF patients included those with pulmonary stenosis (TOF-PS) or double-outlet right ventricle (DORV) variants who underwent either transannular or infundibular patch repair, as well as those with pulmonary atresia (TOF-PA) who underwent RV-to-pulmonary artery (RV-PA) conduit repair. Patients with poor imaging quality or significant stent/sternal artifact were excluded. Patients with evidence of elevated pulmonary vascular resistance (confirmed by cardiac catheterization) were also excluded. For comparison, CMR datasets from normal controls and RV dilation patients were included. RV dilation patients were defined as those with ASDs or partial anomalous pulmonary venous return (PAPVR) with hemodynamically significant shunt (Qp:Qs > 1.2:1). Normal controls included those who underwent CMR imaging for separate clinical indication (exclude cardiomyopathy, atrial shunts or PAPVR) and found to have normal RV size, function and pulmonary-to-systemic flow ratio (Qp:Qs) < 1.2:1.

All studies were performed with a 1.5 T CMR scanner (Siemens Healthineers, Erlangen, German). CMR data included cine imaging (long-axis and short-axis cine), contrast-enhanced magnetic resonance angiography (MRA), three-dimensional (3D) steady state free precession imaging (3D SSFP), two-dimensional phase contrast across the pulmonary valve (Venc set between 2 and 2.5 m/s) and 4D flow. The 4D flow sequence parameters included FOV = 280–480 × 140–230 mm, matrix = 160 × 77, TE = 2.19 ms, TR = 37.9–59.4 ms (dependent on number of segments per RR and RR interval; either 2 segments for RR > 750 ms or 3 segments for RR < 750 ms), flip angle = 15, slice thickness = 1.8 mm or 2.8 mm (dependent on patient size, either 1.8 mm for BSA < 1.5 m^2^; 2.8 mm for BSA > 1.5 m^2^), venc = 2 m/s–2.5 m/s and number of reconstructed phases = 20–30. The MRA and 3D SSFP covered the entire heart with voxel size ~ 1.4 × 1.4 × 1.4 mm. Standard clinical measurements of RVEDVI, RVESVI, RVEF, and PR% were obtained. For rTOF patients, electrocardiogram and cardiopulmonary exercise stress test results within 1 year of CMR study were also collated for QRS duration, VO_2-max_ and % predicted VO_2-max_.

### 3D modeling of RVOT and RV inflow

**Fig. 1:**
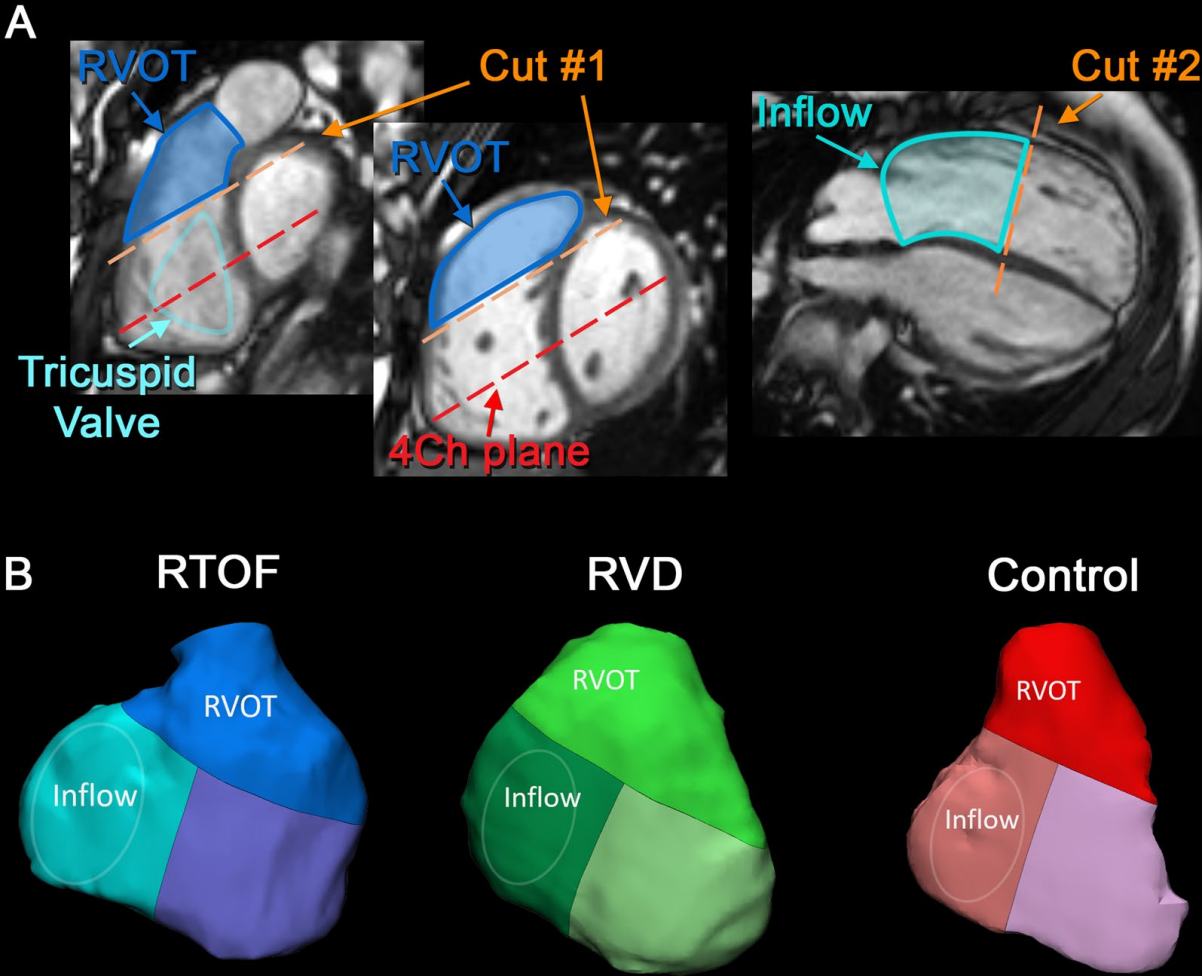
The inflow and outflow components of the right ventricle (RV). Three-dimensional (3D) end-diastolic models of the RV for all three groups were systematically performed to separate out the RV outflow tract (RVOT) and RV inflow. This was performed by Cut #1 and Cut #2 as shown in **A** with corresponding landmarks. The resulting 3D models of the inflow and outflow components are shown in **B**

3D end-diastolic models of the RV were first created from MRA and 3D SSFP datasets, according to lab standard segmentation [21, 22] using commercially available software (Mimics; Materialise, Leuven, Netherlands). The RV was isolated between the tricuspid valve and pulmonary valve annuli. For rTOF with transannular patch, the pulmonary valve annulus was determined by presence of either residual valve sinuses or valve tissue. The 3D models were then consistently divided into separate components of the RV (Fig. 1), accounting for the variable geometry of the RV and RVOT: #1) A dividing plane (parallel to 4-chamber cine plane) at the antero-septal commissure of tricuspid annulus (most cranial aspect) was first used to separate the RVOT and RV body; #2) the remaining RV body was then divided half-way down the long-axis to separate the RV inflow from RV apex. The resulting RVOT volume is in line with corresponding RV segments as described by Wald et al. (which also separated the RV into components) [23].

### Post-processing of 4D flow

Each 4D flow dataset was imported into IT Flow (Cardioflow, Tokyo, Japan) [24] as separate segmentation masks to isolate velocity flow fields within the domains of interest. After background-phase correction, the masks were further screened to remove regions of noise artifact. Particle tracking and vorticity mapping analysis was also performed for qualitative assessment.

### Quantification of vorticity, RVOT vorticity quotient and RV diastolic vorticity quotient

Vorticity was quantified for each phase of the cardiac cycle by IT Flow. Conceptually, vorticity represents the magnitude and the axial direction of spinning. Vorticity is defined as the curl of the velocity field:

$$\overrightarrow{\omega }=\nabla \times \overrightarrow{u}$$

Absolute vorticity (units of L/s) is calculated by spatially integrating the vorticity magnitude $\left\| {\mathop \omega \limits^{ \to } } \right\|$, that accounts for all three directions of spinning, within each voxel:

$$Absolute\;Vorticity = \smallint _{V} \left\| {\vec{\omega }} \right\|dV$$

where V is the segmented volume of interest (e.g. RVOT or inflow region). Since absolute vorticity is dependent on segmentation volume (units of L), the calculated results were normalized against the volume of the segmented region as $\omega$ (units of 1/s):

$$\omega _{{RVOT}} = \frac{{Absolute\;Vorticity_{{RVOT}} }}{{Volume_{{RVOT}} }}$$

$$\omega _{{RVInflow}} = \frac{{Absolute\;Vorticity_{{RVInflow}} }}{{Volume_{{RVInflow}} }}$$

**Fig. 2:**
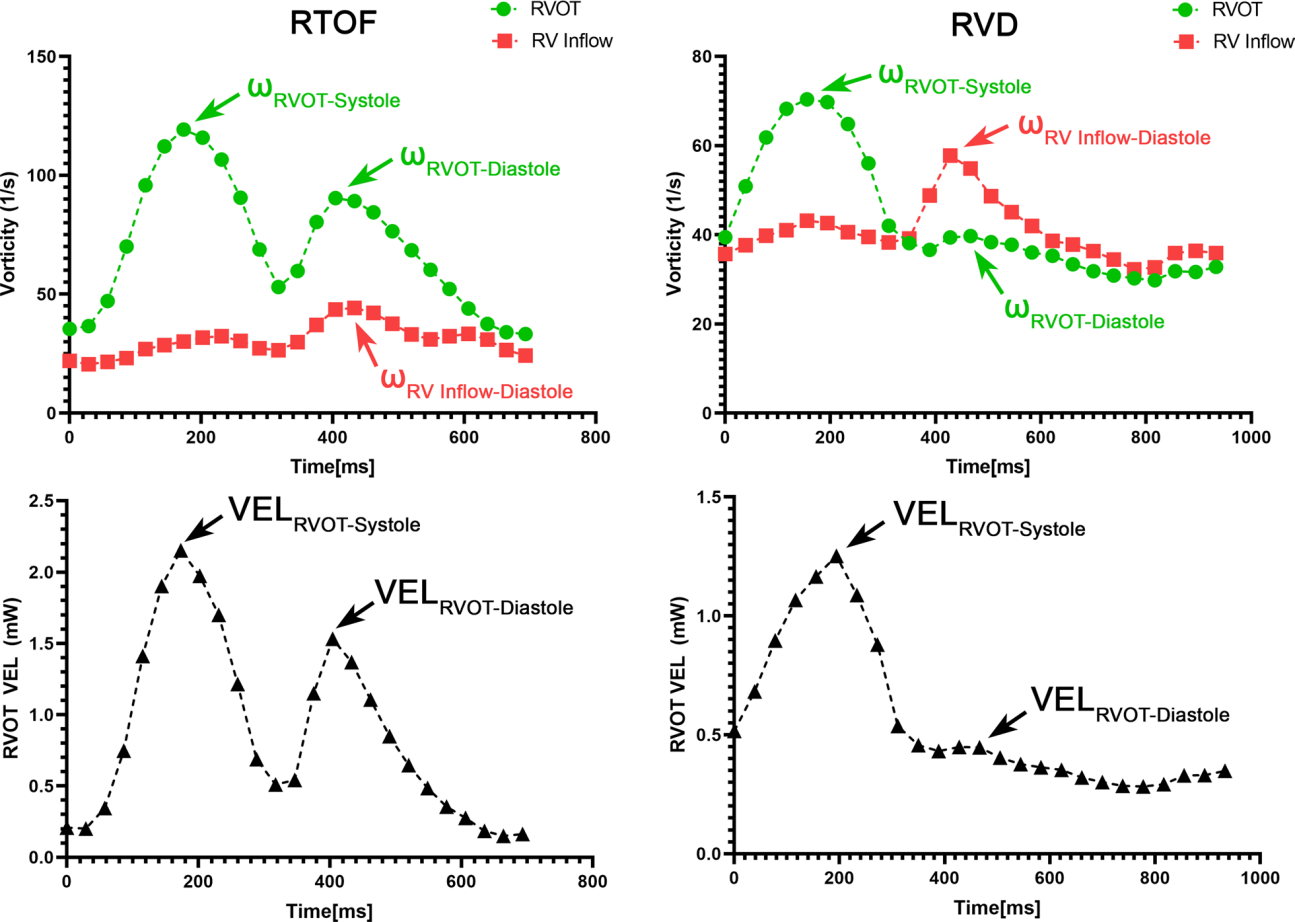
Example vorticity and energy loss profiles in a repaired tetralogy of Fallot (rTOF) and right ventricular dilation (RVD) patient. For each dataset, the peak systolic/diastolic viscous energy loss, systolic/diastolic vorticity in the RV outflow tract (RVOT) and RV inflow was selected

The peak value within the RVOT during systole (ω_RVOT-Systole_), as well as the RVOT and RV inflow during diastole (ω_RVOT-Diastole_, ω_RV Inflow-Diastole_) were measured (Fig. 2). To characterize the relative influence of vorticity between RV inflow vs PR, and remove other confounding effects such as heart rate and voxel size [25], these measurements were normalized into unitless parameters in a similar manner as PR% and echocardiographic Doppler metrics of rTOF [26, 27]. ω_RVOT-Diastole_/ω_RV Inflow-Diastole_ was calculated and represented as the RV Diastolic Vorticity Quotient (RV-DVQ). ω_RVOT-Diastole_/ω_RVOT-Systole_ was also calculated and represented as the RVOT Vorticity Quotient (RVOT-VQ).

### Quantification and analysis of RVOT viscous energy loss, RVOT energy quotient

Viscous energy loss (VEL, mW) represent viscous dissipation across the flow field within each voxel, a combined effect of blood viscosity and velocity gradient (shear) [24]. For each direction, E is summated over repeated indices as:

$$VEL = ~\int {\frac{1}{2}} \mu \sum {\left( {\frac{{\partial u_{i} }}{{\partial x_{j} }} + \frac{{\partial u_{j} }}{{\partial x_{i} }}} \right)^{2} } dV$$

where $\mu$ is blood viscosity at 0.004 Pa·s. The peak VEL across the RVOT in systole (VEL_RVOT-Systole_) and diastole (VEL_RVOT-Diastole_) was measured (Fig. 2). VEL_RVOT-Diastole_/VEL_RVOT-Systole_ was also calculated as the RVOT Energy Quotient (RVOT-EQ).

### Statistical analysis

All statistical analysis was performed with Prism 8 (Graphpad, San Diego, California, USA). One-way analysis of variance (ANOVA) was used to compare between rTOF patients with severe PR (defined as PR% > 40%), rTOF patients with moderate PR (PR% 20–40%), rTOF patients with mild PR (PR% < 20%), RV dilation and control groups. Subgroup analysis of rTOF patients was also performed: #1) rTOF patients with TOF-PS anatomy and only infundibular or transannular patch repair (i.e. no conduit placement) with no further surgical reintervention (i.e. no PVR), reducing any variation to RVOT geometry from a conduit hood or PVR; #2) rTOF patients with exercise stress test results. Correlations between continuous variables were assessed using Pearson’s correlation coefficient [28]. Probability values < 0.05 were considered statistically significant.

## Results

### Demographics

58 rTOF patients, 28 RV dilation patients and 12 normal controls were included (Table 1). rTOF patients consisted of 47 with TOF-PS (2 DORV variants) and 11 with TOF-PA; 42 had transannular patch repair, 7 had infundibular patch repair, and 9 underwent RV-PA conduit repair. 11 rTOF patients were status-post PVR at the time of CMR (Table 2). The mean PR% was 27.9 ± 16.4%, with 20 patients having severe PR, 18 with moderate PR and 20 with mild PR. RV dilation patients consisted of 24 with PAPVR, and 4 with secundum type ASD. Within PAPVR patients, 17 had right-sided PAPVR and associated atrial communication (15 with superior sinus venosus defect, 2 with inferior sinus venosus defect), and the remaining 7 had isolated left-sided PAPVR. There was selection bias in that rTOF patients were older and larger compared to RV dilation and control patients, although both rTOF and RV dilation patients had similar enlarged RVEDV and RVEDVI (203 ± 81 mL and 131 ± 33 mL/m^2^ vs 165 ± 110 mL and 147 ± 58 mL/m^2^, p = ns).

**Table 1:** Overall demographics

	rTOF patients (n = 58)	RV dilation patients (n = 28)	p	Normal controls (n = 12)	p
Demographics					
Age (years), IQR	21.7 (12.2–29.6)	11.5 (4.0–15.5)	< 0.001	13.9 (9.1–17.7)	0.038
Female	30 (51%)	14 (50%)		6 (50%)	
BSA (m^2^)	1.5 ± 0.40	1.2 ± 0.6	< 0.001	1.4 ± 0.5	ns
Native anatomy					
	TOF-PS 47 (84%) TOF-PA 11 (16%)	PAPVR 24 (85%) 2° ASD 4 (15%)			
CMR data					
RVEDV (mL)	203 ± 81	165 ± 110	ns	131 ± 55	0.0017
RVEDVI (mL/m^2^)	131 ± 33	148 ± 57	ns	86 ± 15	< 0.001
RVESVI (mL/m^2^)	65 ± 21	61 ± 29	ns	38 ± 10	< 0.001
RVEF (%)	51.1% ± 5.5%	60.4% ± 6.1%	< 0.001	60.0% ± 5.8%	< 0.001
PR%	27.9% ± 16.4%	0.0 ± 0.04%	< 0.001	0.0 ± 0.03%	< 0.001
Qp:Qs	1.0 ± 0.05	2.5 ± 1.4	< 0.001	1.0 ± 0.08	ns

Fifty-eight repaired tetralogy of Fallot (rTOF) patients, 28 patients with right ventricular (RV) dilation and twelve normal controls were included. RV dilation patients had either partial anomalous pulmonary venous return (PAPVR) or large secundum type atrial septal defects (2° ASD). There was selection bias in that rTOF patients tended to be older and larger than RV dilation patients, although had similar indexed right ventricular end-diastolic volume index (RVEDVI) and RV end-systolic volume index (RVESVI)

*BSA* body surface area, *RVEF* right ventricular ejection fraction, *PR%* pulmonary regurgitation percent

**Table 2:** Demographics of repaired tetralogy of Fallot (rTOF) cohort

	TOF-PS (n = 47)	TOF-PA (n = 11)
Age of complete repair (years), IQR	1.4 (0.3–1.9)	1.2 (0.2–1.7)
Type of surgery		
Initial Blalock-Taussig-Thomas shunt palliation	8 (17%)	3 (27%)
Transannular patch	39 (83%)	3 (27%)
Infundibular patch	7 (15%)	0 (0%)
Right ventricle-to-pulmonary artery conduit	1 (2%)	8 (72%)
Subsequent pulmonary valve replacement	8 by surgery (17%)	2 by surgery, 1 by catheterization (27%)

The cohort consisted of patients with either tetralogy of Fallot-Pulmonary Stenosis (TOF-PS) or tetralogy of Fallot-Pulmonary Atresia (TOF-PA). 10 patients already had pulmonary valve replacement

### Comparison of vorticity between rTOF, RV dilation and control

**Fig. 3:**
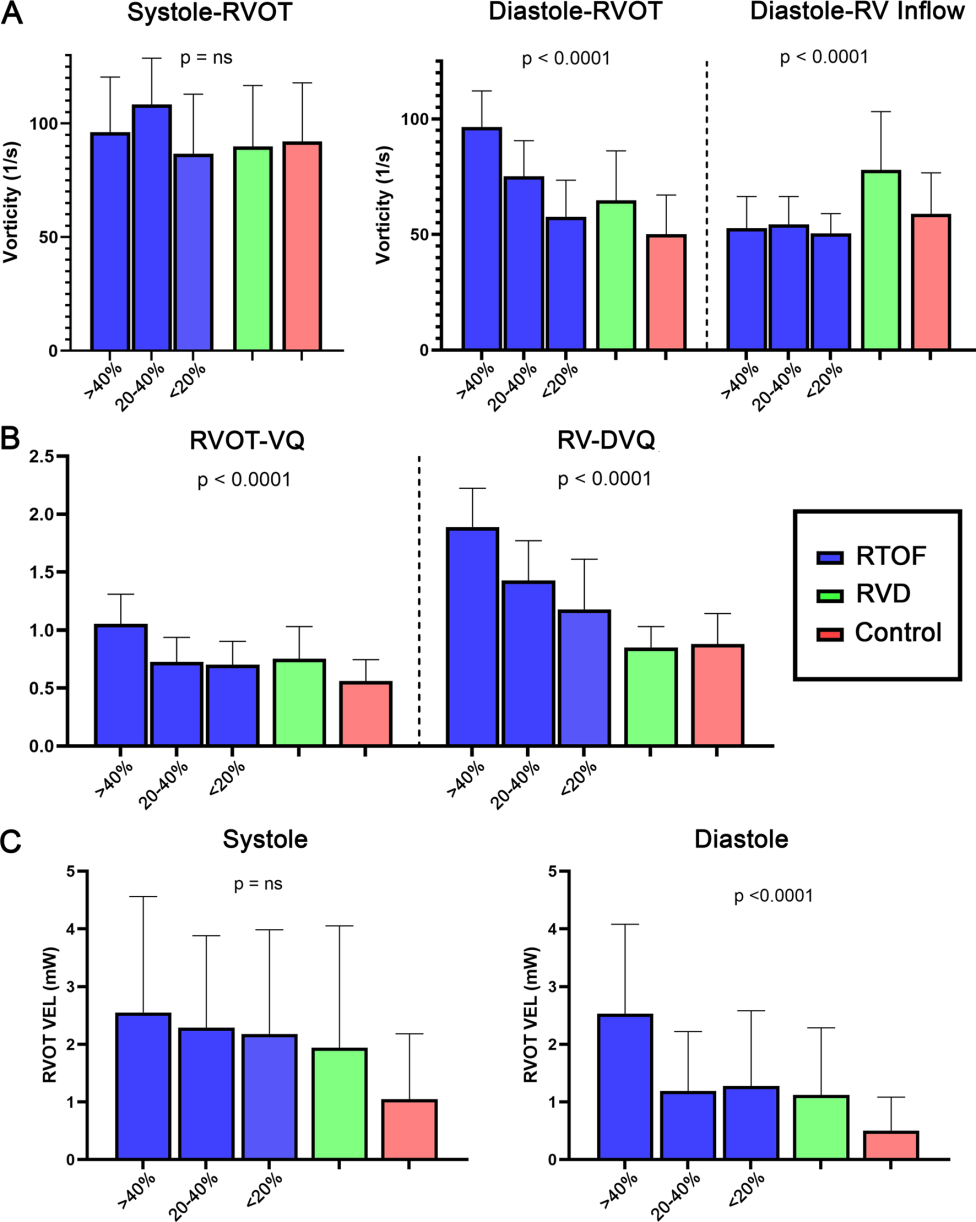
Vorticity and power loss results. When compared to  right ventricular dilation (RVD) and control groups, repaired tetralogy of Fallot (rTOF) patients with severe pulmonary regurgitation (PR% > 40%) had the highest diastolic vorticity in the right ventricular outflow tract (RVOT), as shown in **A**. This group also had the highest diastolic vorticity quotient (RV-DVQ) and right ventricular outflow tract vorticity quotient (RVOT-VQ) as shown in **B**; as well as the highest  RVOT viscous energy loss (VEL) as shown in **C**

Overall quantification results of vorticity are shown in Fig. 3A, B. Between rTOF, RV dilation and control groups, there were no significant differences in regional systolic vorticity (p = ns). Meanwhile, ω_RVOT-Diastole,_ ω_RV Inflow-Diastole_, RVOT-VQ and RV-DVQ were different across groups (p < 0.001). ω_RVOT-Diastole_ and RVOT-VQ were highest in rTOF with severe PR (96.5 ± 15.6/s, 1.05 ± 0.26) compared to rTOF with moderate PR (75.3 ± 15.3/s, 0.73 ± 0.21), rTOF with mild PR (57.6 ± 15.9/s, 0.70 ± 0.20), RV dilation (64.7 ± 21.5/s, 0.75 ± 0.28) and normal controls (50.2 ± 16.9/s, 0.56 ± 0.18). ω_Inflow-Diastole_ was highest in RV dilation patients (77.9 ± 25.3/s), compared to rTOF patient with severe PR (52.7 ± 13.6/s), rTOF patients with moderate PR (54.5 ± 12.0/s), rTOF with mild PR (50.4 ± 8.6/s), and control group (58.9 ± 17.8/s). RV-DVQ was highest in rTOF with severe PR (1.88 ± 0.33), compared to rTOF with moderate PR (1.43 ± 0.34) and rTOF with mild PR (1.17 ± 0.43); whereas RV-DVQ is normalized in RV dilation patients (0.85 ± 0.18) and similar to normal controls (0.87 ± 0.26).

### Comparison of RVOT energy loss between rTOF, RV dilation and control

VEL measurements across the RVOT are demonstrated in Fig. 3C. There were no significant differences in VEL_RVOT-Systole_ between rTOF, RV dilation and control groups (p = ns). Meanwhile, there were differences in VEL_RVOT-Diastole_ and RVOT-EQ (p < 0.001). VEL_RVOT-Diastole_ and RVOT-EQ was highest in rTOF with severe PR (2.53 ± 1.51 mW, 1.24 ± 0.65) compared to rTOF with moderate PR (1.18 ± 1.03 mW, 0.56 ± 0.31), rTOF with mild PR (1.27 ± 1.31 mW, 0.55 ± 0.24), RV dilation patients (1.12 ± 1.16 mW, 0.75 ± 0.28) and normal controls (0.50 ± 0.58 mW, 0.56 ± 0.18).

### Qualitative comparison of intracardiac flow

**Fig. 4:**
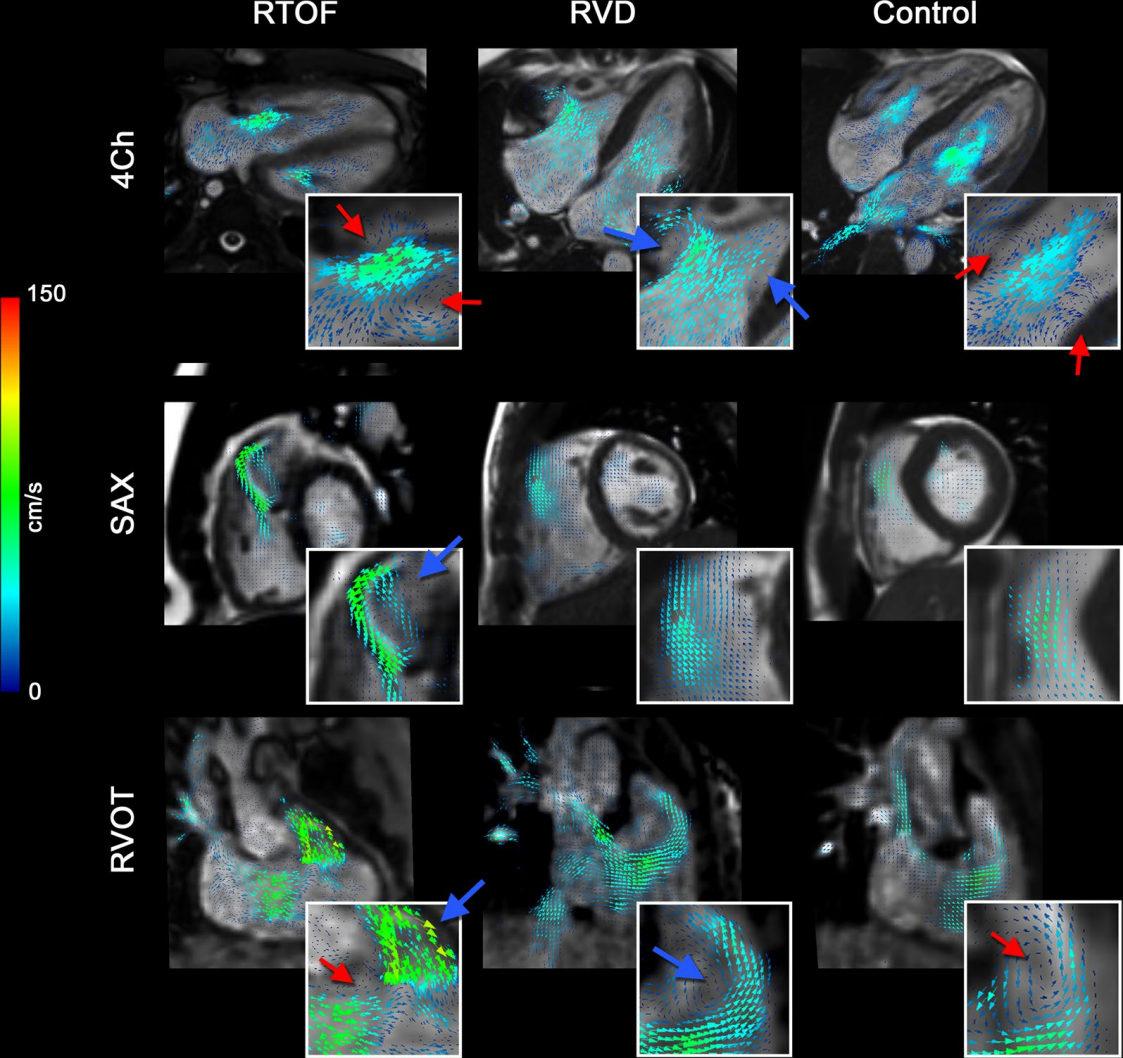
Qualitative intracardiac flow profile via velocity vector fields overlaid onto cine imaging. Blue arrows are pointed to larger vorticity magnitude, red arrows pointed to smaller vorticity magnitude. In rTOF patients, there is a dominant vortex generated from pulmonary insufficiency, best visualized in short axis (SAx) and modified right ventricular outflow tract (RVOT) view. This vortex is separate from the smaller vortices generated by the tricuspid inflow, best visualized in the 4-chamber (4Ch) view. For right ventricular dilation (RVD patients), a larger vortex is generated from tricuspid inflow and propagates into the RVOT, but the overall flow topology is unchanged when compared to controls

**Fig. 5:**
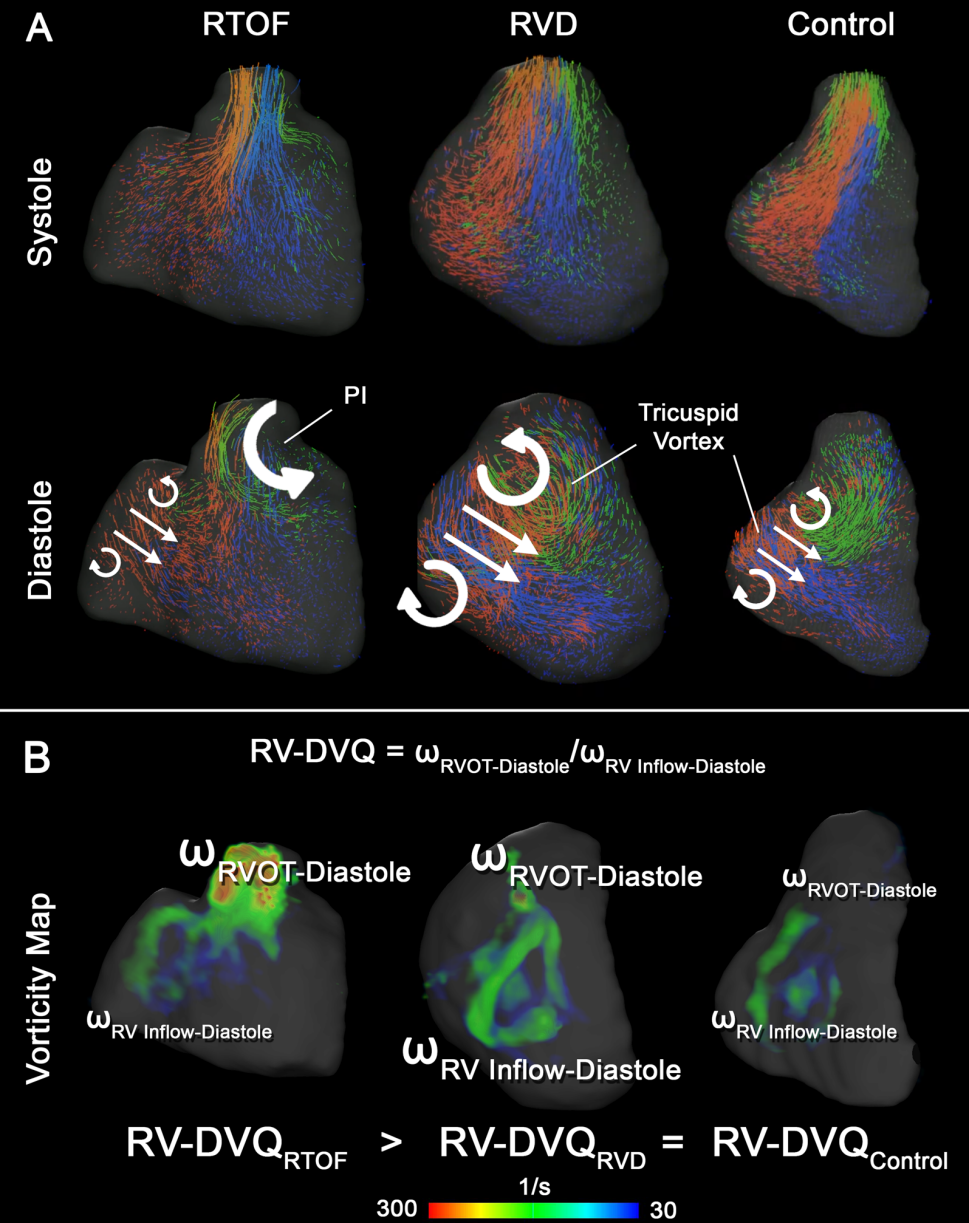
Qualitative intracardiac flow profile via particle tracking (**A**, **B**) vorticity mapping. **A** Particles were color-coded based on original position in end-diastole. Red particles start from tricuspid inflow, green particles from right ventricular outflow tract (RVOT), and blue from remaining body/apex. An organized, “donut”-shaped ring-vortex was visualized surrounding the tricuspid inflow in right ventricular dilation (RVD) and control cases, whereas this profile is disrupted in repaired tetralogy of Fallot (rTOF) patients. **B** Volumetric representation of vorticity also demonstrates the ring-vortex as shown. The calculation of RV Diastolic Vorticity Quotient (RV-DVQ) in RTOF patients demonstrates the influence of vorticity from RVOT; whereas in RVD and control cases, vorticity from RV inflow remains dominant

The representative differences in flow topology between each cohort are demonstrated by velocity vector fields (Fig. 4), particle tracking (Fig. 5A) and vorticity mapping (Fig. 5B). During systole, the particle tracking profile appeared similar between groups. In diastole for both RV dilation and control cases, an organized, “donut”-shaped ring-vortex was visualized surrounding the tricuspid inflow. The superior edge of the diastolic vortex propagated in a counterclockwise pattern directly towards the RVOT. In comparison, the rTOF cohort in diastole demonstrated dominant vorticity formation starting in the RVOT from PR, limiting the ring-vortex profile from tricuspid valve.

### Subgroup analysis of TOF-PS patients and only primary repair

Among the rTOF cohort, there were 36 patients who had TOF-PS status-post infundibular or transannular patch repair with no further surgical reintervention. Within this subgroup, there were fair-to-high correlations between intracardiac flow parameters with RVEDVI, RVESVI, RVEF and PR% (Table 3, Fig. 5A–C). RV-DVQ correlated with RVEF (− 0.521, p = 0.001), PR% (0.774, p < 0.001), RVEDVI (0.683, p < 0.001), RVESVI (0.696, p < 0.001) and QRS duration (0.402, p = 0.017). RTOF-EQ correlated with RVEF (− 0.472, p = 0.0032), PR% (0.584, p <  0.001), RVEDVI (0.415, p = 0.011), RVESVI (0.461, p = 0.004).

**Table 3:** Subgroup correlation analysis of repaired tetralogy of Fallot (rTOF) patients who had infundibular or transannular patch repair (i.e. no conduit placement) and no pulmonary valve replacement (n = 36)

	RVEF %	p	PR %	p	RVEDVI	p	RVESVI	p	QRS	P
Conventional										
RVEF (%)			− 0.460	0.0048	− 0.453	0.005	− 0.685	< 0.001	− 0.408	0.015
PR%	− 0.460	0.005			0.702	< 0.001	0.695	< 0.001	0.408	0.017
RVEDVI (mL/m^2^)	− 0.453	0.005	0.702	< 0.001			0.956	< 0.001	0.421	0.012
RVESVI (mL/m^2^)	− 0.685	< 0.001	0.695	< 0.001	0.956	< 0.001			0.449	0.007
QRS (ms)	− 0.4075	0.015	0.4076	0.017	0.4211	0.012	0.4498	0.007		
Vorticity										
ω_RVOT-Diastole_	− 0.327	0.048	0.794	< 0.001	0.637	< 0.001	0.599	< 0.001	0.247	ns
RTOF-VQ (ω_RVOT-Diastole_/ω_RVOT-Systole_)	− 0.496	0.002	0.7390	< 0.001	0.635	< 0.001	0.644	< 0.001	0.313	ns
RV-DVQ (ω_RVOT-Diastole_/ω_RV Inflow-Diastole_)	− 0.521	0.001	0.774	< 0.001	0.683	< 0.001	0.696	< 0.001	0.402	0.017
Energy loss										
VEL_RVOT-Diastole_	− 0.373	0.023	0.607	< 0.001	0.747	< 0.001	0.721	< 0.001	0.392	0.020
RTOF-EQ (VEL_RVOT-Diastole_/VEL_RVOT-Systole_)	− 0.472	0.003	0.584	< 0.001	0.415	0.011	0.461	0.004	0.201	ns

### Subgroup analysis of rTOF patients with exercise stress test

**Fig. 6:**
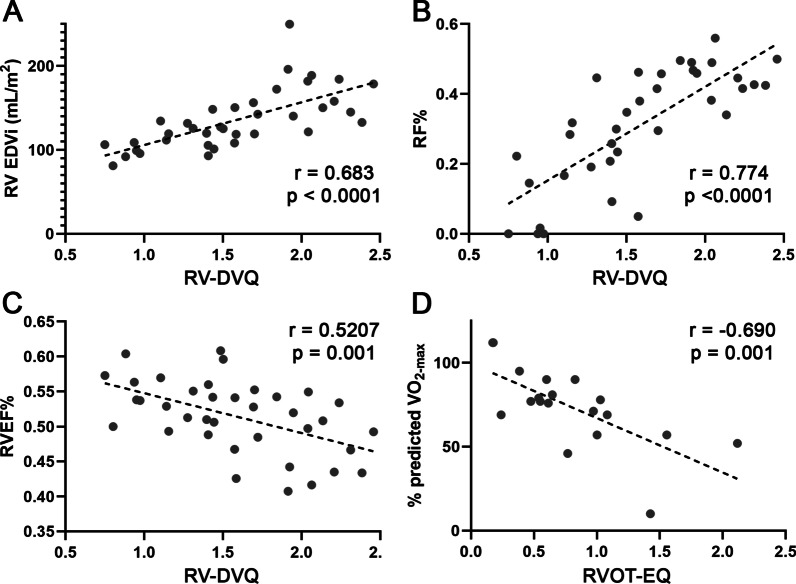
Correlations of vorticity and energy loss to conventional indices in subgroup analysis. **A**–**C** are from subgroup analysis of repaired tetralogy of Fallot (rTOF) patients with previous anatomy of Tetralogy of Fallot, Pulmonary stenosis (TOF-PS) and only infundibular or transannular patch repair (i.e. no conduit placement) and no further surgical reintervention (i.e. no pulmonary valve replacement). **D** is from subgroup analysis of RTOF patients with exercise stress test

Among the rTOF cohort, 19 patients had exercise stress test performed within 2.1 ± 5.4 months of CMR study. This subgroup cohort consisted of 16 with TOF-PS and 3 with TOF-PA; 17 had transannular patch, 2 had conduit repair. 7 patients already had PVR. The mean PR% was 30 ± 16%. The mean VO_2-max_ and % predicted VO_2-max_ was 27.4 ± 7.72 mL/kg/min and 73.6 ± 23.5% respectively. There were no significant correlations between VO_2-max_ and % predicted VO_2-max_ with conventional CMR measurements of the RV. Meanwhile, exercise stress test results moderately correlated with 4D flow parameters (Table 4, Fig. 6D). RVOT-VQ and RVOT-EQ negatively correlated with VO_2-max_ (− 0.587, p = 0.008; -0.617, p = 0.005) and % predicted VO_2-max_ (− 0.678, p = 0.0014; − 0.690, p = 0.001).

**Table 4:** Subgroup correlation analysis of repaired retralogy of Fallot (rTOF) patients with exercise stress test results (n = 19)

	VO_2-max_	p	% Predicted VO_2-max_	p
Conventional				
RVEF (%)	− 0.291	ns	0.259	ns
PR%	− 0.192	ns	− 0.267	ns
RVEDVI (mL/m^2^)	0.252	ns	− 0.046	ns
RVESVI (mL/m^2^)	0.313	ns	− 0.166	ns
QRS (ms)	− 0.235	ns	− 0.069	ns
Vorticity				
ω_RVOT-Diastole_	− 0.434	ns	− 0.5497	0.016
RVOT-VQ (ω_RVOT-Diastole_/ω_RVOT-Systole_)	− 0.587	0.008	− 0.678	0.001
RV-DVQ (ω_RVOT-Diastole_/ω_RV Inflow-Diastole_)	− 0.266	Ns	− 0.641	0.003
Energy Loss				
VEL_RVOT-Diastole_	− 0.497	0.03	− 0.375	ns
RVOT-EQ (VEL_RVOT-Diastole_/VEL_RVOT-Systole_)	− 0.617	0.005	− 0.6902	0.001

## Discussion

This study retrospectively analyzed 4D flow datasets to quantify regional vorticity and energy loss in rTOF patients, compared to normal normal controls and RV dilation patients. The main findings of this study include: (1) increased RVOT vorticity and energy loss in diastole in rTOF patients, whereas diastolic vorticity in RV inflow remained dominant in normal controls and RV dilation patients; (2) correlation of RVOT vorticity and energy loss with RV dysfunction in TOF-PS patients; and (3) correlation of RVOT vorticity and energy loss with exercise intolerance.

Conventional CMR measurements inform the timing of PVR, albeit with limitations. The timing of PVR should be optimized to prevent long-term, irreversible consequences of RV dysfunction such as ventricular arrhythmias, exercise intolerance and overt systolic dysfunction [6, 29–31]. To date the International Multicenter TOF Registry (INDICATOR), a multi-center international cohort study, provides the best interpretation of RV dysfunction in rTOF patients by CMR, identifying RVEF as a significant risk factor for death and ventricular tachycardia [32]. However, it is also clear that conventional metrics of PR% and ventricular size/function cannot predict deterioration of the RV [4]. It has also been shown by meta-analysis that for large RVs, improvements in RV size after PVR do not necessarily improve RVEF and is less correlated to improvement of symptoms [33]. Electromechanical dyssynchrony and scar burden appear to be more associated with exercise intolerance and RVEF compared to PR% [10, 34]. The CMR metrics do not fully capture the underlying pathophysiology of RV dysfunction in rTOF patients. Thus, PR and RVEDVI are only fairly correlated with RVEF and not correlated with exercise capacity. Meanwhile, the proposed 4D flow parameters have correlations with RVEF, RF%, RVEDVI, and exercise capacity.

The 4D flow parameters and analysis of diastolic vortical flow patterns show that RV dilation patients still have preserved diastolic filling, despite having larger RV size. In control and RV dilation cases, the observed “donut” of diastolic vortex from tricuspid inflow is consistent with our previous *in-vitro* investigations [18–20], computational studies derived from 3D echocardiography [35–37] and in-vivo observations of 4D flow [38]. Early work by Pasipoularides et al. demonstrated the role of the diastolic vortex in reducing the “convective deceleration load” and improve ventricular filling [36]. Specifically, the “donut” vortex is likely generated by the longitudinal motion of the RV during diastole, with no active acceleration/deceleration components that would exert additional hemodynamic forces in the RV. The propagation of the inflow vortex towards the RVOT may also facilitate systolic flow [39]. RV-DVQ takes into account the relative impact of the inflow vortex towards the RVOT; thus as a normalized parameter, RV-DVQ is not significantly different between RV dilation and normal controls (Fig. 4B).

In our study, specific regional measurement by 4D flow was key to highlighting the complex interplay between PR and RV size/function in rTOF patients. *In-vivo* and *in-vitro* studies of aortic regurgitation in the left ventricle show that when the inflow vortex is interrupted by a colliding jet of insufficiency, there is increased vortical flow formation and energy loss [40–42]. rTOF patients in similar fashion have higher vorticity and energy loss from PR in the RVOT, as the local jet collision creates a distinct intracardiac topology which generates shear layers, small-scale turbulence or turbulent-like flow [16, 18–20]. This effect is determined by PR, inflow, RV geometry (e.g. the relative angle of inflow and PR), which may explain why RVOT vorticity and energy loss correlate with RV size, RV function and PI in RTOF. Considering that RV function and exercise capacity tend to be preserved in rTOF with transatrial-transpulmonary approach, or in patients with isolated PS status post valvotomy (i.e. minimized or absent ventriculotomy in RVOT) [43, 44], the RVOT may also function to attenuate the abnormal flow features from PR.

Furthermore, the native diastolic vortex from tricuspid inflow is dominated by RVOT vorticity, leading to higher RV-DVQ (Fig. 4B). RV-DVQ was also correlated with longer QRS duration (i.e. right bundle branch block). Electromechanical dyssynchrony likely affected RV longitudinal wall motion [10], which in turn weakens the tricuspid inflow vortex. The competing nature between PI and tricuspid inflow is also observed in *in-vitro* studies [16, 18], and may lead to abnormal hemodynamic forces in directed from the RVOT towards the RV base, as demonstrated by Sjoberg et al. [45]. The vortex interaction and resultant hemodynamic forces likely contributes to altered mechanotransductive environment that leads to RV dysfunction [46, 47]. The abnormal hemodynamic forces may also contribute to the abnormal RVOT shape geometry found in larger RVs [7]. PVR would likely restore the natural diastolic vortex and help restore RV function; thus RVOT vorticity and energy loss could potentially be biomarkers to guide PVR therapy.

The subgroup correlation results of RV-DVQ, RVOT-VQ and RVOT-EQ suggest that the 4D flow measurements, by capturing PR as localized intracardiac phenomena, reflects the clinical range of RV dilation and dysfunction created in rTOF patients. Shibata et al. using vector flow mapping by echocardiography, demonstrated that diastolic energy losses correlated with RV deterioration [48]. Measurements of kinetic energy have also demonstrated global RV abnormalities with varying correlations to RV size and RV function [15, 49]. Frediksson et al. in particular measured turbulent kinetic energy [14] derived from the magnitude imaging of individual flow-encoding segments rather than the velocity field. In that study, rTOF patients with larger RVEDVI had higher turbulent kinetic energy. These abnormalities are likely sources of energy loss that contributes to RV pump inefficiency in rTOF patients [48, 50].

Furthermore, the interaction between PR and tricuspid inflow potentially limits augmentation of RV cardiac output during exercise, leading to exercise intolerance. Previous CMR studies have not demonstrated any significant correlation between RV size and PR with exercise capacity [8, 9, 11], informing the use of modified parameters such as ventricular global function index [9]. Thus far, only right ventricular systolic dysfunction and branch pulmonary artery size discrepancy have correlated with decreased exercise capacity [7, 11, 51]. In our subgroup cohort, RVOT-VQ and RVOT-EQ correlated with VO_2-max_ and % predicted VO_2-max_, suggesting that the abnormal intracardiac flow patterns from PR is still the link to exercise intolerance in rTOF patients. Ideally, these 4D flow parameters would be investigated with exercise CMR [52], although the decreased signal-to-noise ratio and motion artifact during exercise would likely limit interpretation of such findings. We aim to develop these as novel imaging biomarkers linking the pathophysiology between PR and exercise intolerance, to ultimately assist in the decision of optimal timing of PVR.

In addition to intracardiac flow, there are other potential biomarkers of RV size/function beyond volumetric measurements. Studies investigating myocardial strain by feature tracking have demonstrated impairments in RV global strain and ventricular dyssynchrony in rTOF patients [30, 53]. RV scar burden as defined by late gadolinium enhancement volume also correlates with ventricular dysfunction and risk of inducible ventricular arrhythmias [34]. Shape modeling studies have demonstrated morphological changes beyond measurements of RVEDVI and RVESVI [5, 7, 54]. Computational load analysis of the RV myocardium, including the akinetic RVOT free wall [55] may also be sensitive in predicting RV dysfunction.

### Prior studies

Compared to other 4D CMR studies on rTOF, our study has the benefit of (1) a larger sample size, with comparison against normal controls as well as RVD representing other chronic volume loading states and (2) a specific regional focus of 4D flow biomarkers to avoid dilution of results by including tricuspid inflow. For example, Hirtler et al. investigated 24 rTOF patients and measured higher mean RV vorticity, but these measurements were paradoxically negatively correlated with RV size [12]. This finding likely resulted from measurements derived in the 4-chamber cine plane, which only isolates the tricuspid inflow vortex (whereas our data would suggest that this component is weakened by PR).

### Study limitations

The study, while larger than other 4D flow studies of rTOF, is still a single-center analysis and limited by sample size. The rTOF cohort was heterogenous in both anatomy and surgical repair, limiting the correlations observed in the study (although subgroup analysis of TOF-PS patients still had consistent results). As a retrospective study, the clinical 4D flow datasets did not include dedicated magnitude imaging which is required to calculate other 4D flow-based parameters such as turbulent kinetic energy. Late gadolinium enhancement was also not routinely performed in this study, which precluded the investigation of peri-patch fibrosis in the RVOT [34] and its effect on intracardiac flow. For 4D flow analysis, we intentionally cut arbitrary, static planes in RV end-diastolic models to minimize inter-observer variability inherent with mask segmentation of RV regions. This potentially limited analysis of the RVOT during systolic movement, although this effect may be minimal as rTOF cases tend to have akinetic RVOT. There was inconsistent timing of exercise stress test in rTOF patients, although the time interval between CMR and stress tests were still within recommended surveillance guidelines of rTOF patients [2] and is unlikely to significantly alter results based on previous longitudinal studies [56, 57]. Most importantly, the limited spatial and temporal resolution of 4D flow prohibited detailed assessment of smaller vortical interactions formed by the collision of PR into the tricuspid vortex and the propagation of turbulent flow into the RV body and myocardium, particularly the trabeculations [58]. Thus, we did not assess hemodynamic parameters such as wall shear stress along the RV free wall, which likely has an influence on RV dilation.

### Future studies

Future work will focus on in-depth flow quantification in rTOF patients, to further elaborate on the influence of vorticity/energy loss towards RV dysfunction and exercise intolerance. These will include refining computational models of RV intracardiac flow in rTOF patients [18–20] and using advanced echocardiography techniques such as vector flow mapping [48] or blood speckle imaging [59]. These methods could potentially overcome the spatial and temporal limitations of 4D flow, further refining flow biomarkers to account for other factors such as heart rate, voxel size or presence of late gadolinium enhancement. As the variable RVOT geometry in rTOF patients [7] may also alter the angle of jet collision between PI and tricuspid inflow, we will also investigate this relationship with statistical shape modeling. We plan to prospectively use 4D flow and computational modelling to investigate intracardiac flow in RTOF patients before-and-after PVR, as well as during exercise. We aim to further develop vorticity and energy loss as clinical biomarkers to aid in timing of PVR for rTOF patients.

## Conclusion

In rTOF patients, abnormal vorticity and energy loss dominate the RVOT compared to tricuspid inflow. These 4D flow-based parameters are potential biomarkers that are distinct from other forms of chronic RV volume loading, representing abnormal intracardiac flow that contribute to RV dysfunction and exercise intolerance. Future studies should investigate the role of intracardiac 4D flow in guiding PVR therapy.

## Data Availability

The datasets used and/or analyzed during the current study are available from the corresponding author on reasonable request.

## Abbreviations

3D: Three dimensional
ASD: Atrial septal defect
CMR: Cardiovascular magnetic resonance
DORV: Double outlet right ventricle
MRA: Magnetic resonance angiography
PAPVR: Partial anomalous pulmonary venous return
PR: Pulmonary regurgitation
PR%: Pulmonary regurgitant fraction
PS: Pulmonic stenosis
PVR: Pulmonary valve replacement
rTOF: Repaired Tetralogy of Fallot
RV: Right ventricle/right ventricular
RV-DVQ: Right ventricular diastolic vorticity quotient
RV-PA: Right ventricle-to-pulmonary artery conduit repair
RVD: Right ventricular dilation
RVEDVI: Right ventricular end-diastolic volume index
RVEF: Right ventricular ejection fraction
RVESVI: Right ventricular end-systolic volume index
RVOT: Right ventricular outflow tract
RVOT-DVQ: Right ventricular outflow tract diastolic vorticity quotient
RVOT-EQ: Right ventricular outflow tract energy quotient
RVOT-VQ: Right ventricular outflow tract vorticity quotient
SAx: Short axis
SSFP: Steady state free precession
TOF-PA: Tetralogy of Fallot with pulmonary atresia
TOF-PS: Tetralogy of Fallot with pulmonary stenosis
VEL: Viscous energy loss
